# Primary care utilisation patterns among an urban immigrant population in the Spanish National Health System

**DOI:** 10.1186/1471-2458-11-432

**Published:** 2011-06-06

**Authors:** Amaia Calderón-Larrañaga, Luis A Gimeno-Feliu, Rosa Macipe-Costa, Beatriz Poblador-Plou, Daniel Bordonaba-Bosque, Alexandra Prados-Torres

**Affiliations:** 1 Instituto Aragonés de Ciencias de la Salud (I+CS), Instituto de Investigación Sanitaria Aragón (IIS Aragón), Zaragoza, Spain; 2 San Pablo Primary Health Care Centre, Servicio Aragonés de Salud (SALUD), Zaragoza, Spain; 3 Fuentes de Ebro Primary Health Care Centre, Servicio Aragonés de Salud (SALUD), Zaragoza, Spain

## Abstract

**Background:**

There is evidence suggesting that the use of health services is lower among immigrants after adjusting for age and sex. This study takes a step forward to compare primary care (PC) utilisation patterns between immigrants and the native population with regard to their morbidity burden.

**Methods:**

This retrospective, observational study looked at 69,067 individuals representing the entire population assigned to three urban PC centres in the city of Zaragoza (Aragon, Spain). Poisson models were applied to determine the number of annual PC consultations per individual based on immigration status. All models were first adjusted for age and sex and then for age, sex and case mix (ACG System^®^).

**Results:**

The age and sex adjusted mean number of total annual consultations was lower among the immigrant population (children: IRR = 0.79, p < 0.05; adults: IRR = 0.73, p < 0.05). After adjusting for morbidity burden, this difference decreased among children (IRR = 0.94, p < 0.05) and disappeared among adults (IRR = 1.00). Further analysis considering the PC health service and type of visit revealed higher usage of routine diagnostic tests among immigrant children (IRR = 1.77, p < 0.05) and a higher usage of emergency services among the immigrant adult population (IRR = 1.2, p < 0.05) after adjusting for age, sex and case mix.

**Conclusions:**

Although immigrants make lower use of PC services than the native population after adjusting the consultation rate for age and sex, these differences decrease significantly when considering their morbidity burden. These results reinforce the 'healthy migration effect' and discount the existence of differences in PC utilisation patterns between the immigrant and native populations in Spain.

## Background

The recent increase in the arrival of immigrants to Spain has engendered a deep interest in studying the implications of this phenomenon on the utilisation of health services. Contrary to the public's prejudice and in agreement with the reality in other countries with longstanding experience in dealing with immigration, there is evidence to suggest that the use of healthcare services is lower among immigrants, even when accounting for age and sex [1]. These striking findings have aroused concerns among certain researchers as to why these differences in healthcare delivery exist.

These inequalities have been attributed to a number of different hypotheses, including difficulties accessing the health system [2], cultural and sociological differences in the perception of illness [3] and better health status of the immigrant population [4]. This study focuses on this last hypothesis. It has been established that the morbidity burden accounts for much of the variation in the consumption of healthcare resources [5], and it warrants further study. Furthermore, this variable must be taken into account when discussing potential inequalities in healthcare delivery.

Most studies have assessed the health status of immigrants based on health surveys. The major limitations of health surveys include the use of non-validated, self-declared data that may differ from the objective health of individuals, a lack of validity of the questions used to classify subjects according to health status, and possible sampling and response biases. On the other hand, electronic medical records offer an excellent opportunity to study the type and burden of morbidity in population subgroups at the individual level [6]. Based on these data, the Adjusted Clinical Groups (ACG) System developed at Johns Hopkins University (Baltimore, MD, USA) comprehensively and longitudinally characterises and describes the case mix of a reference population [7,8].

The Spanish National Health System is a tax-based system which offers universal coverage and where primary care (PC) has gatekeeping functions. As a result, 98% of the general population is assigned to PC centres belonging to the public network [9]. In this context, data extracted from PC medical records held by general practitioners (GPs) allow researchers to carry out population-based studies that include both children and adults.

Concerning immigrants' access to health services, Spain has a rather unique policy considering the European context. Since 2000, even illegal immigrants have been entitled to public health care as long as they meet one of the following conditions: registration with their municipal census (which has no implication on their illegal status), visiting an emergency room, being 18 years old or under, and being pregnant [10]. This particularity of the system, apart from favouring especially vulnerable immigrant groups, decreases the potential selection bias due to a lack of inclusion of those immigrants who do not comply with all legal requirements.

The objective of this study was to compare PC utilisation patterns between immigrants and the native population with regard to their morbidity burden in order to contribute to evidence-based decision making.

## Methods

This retrospective, observational study evaluated 69,067 individuals representing the entire population assigned to three urban PC centres located in those neighbourhoods with the highest immigrant concentration in the city of Zaragoza. Data were obtained from electronic medical records of the Aragon Health Care System (year 2007) after an official request and authorisation. Personal information was made anonymous, and the study was approved by the Ethics Committee for Clinical Investigation of Aragon (CEICA).

Immigrant status was defined as having a foreign nationality [11], and this information was obtained from health insurance cards. In Spain, all immigrants have the right to request a health card identifier regardless of their legal status and provided that they are registered in the local population census. By matching personal identification codes on health insurance cards with those on GPs' electronic medical records, information regarding demographic variables (e.g., age and sex), number of annual visits to the PC centre, and morbidity burden was extracted for each individual. In assessing the morbidity burden, all diagnoses and reasons for visits registered during the study period were coded according to the International Classification of Primary Care (ICPC) [12]. Subsequent conversion ('mapping') was made from the ICPC to the International Disease Classification (ICD-9-CM) [13].

Based on the variables of age, sex and diagnoses registered in the study year, a single ACG category was assigned to each individual [14]. The ACG System (version 8.1^®^) classifies all ICD-9-CM codes based on the following criteria: the duration of the condition (e.g., acute, recurrent, or chronic), severity of the condition (e.g., minor and stable versus major and unstable), diagnostic certainty (symptoms versus documented disease), aetiology of the condition (e.g., infectious, injury, or other), and specialty care involvement (e.g., medical, surgical, obstetrical, or haematological). As a result, individuals within a given ACG have experienced a similar pattern of morbidity and resource utilisation over a given year. For the sake of parsimony, ACGs with a similar expected use of resources are aggregated into so-called Resource Utilisation Bands (RUB 0 = non users, RUB 1 = healthy users, RUB 2 = low morbidity, RUB 3 = moderate morbidity, RUB 4 = high morbidity, and RUB 5 = very high morbidity). Thus, each individual is additionally assigned a RUB category.

### Statistical analysis

A descriptive analysis was first carried out in which the mean and the variance of the continuous variables and the distribution of frequencies of the categorical variables were calculated. The study variable "number of consultations per individual and per year" was classified according to the attended PC health service (e.g., family medicine, paediatrics, nursery, physiotherapy, social work, odontostomatology, midwifery or diagnostic tests) and the type of visit (e.g., demanded visit, emergency visit, programmed visit or home visit).

The sex and age adjusted mean number of consultations was calculated through direct standardisation, taking the population structure of the region into account. Poisson models were applied to determine the number of annual consultations per individual based on immigration status. All models were first adjusted for age and sex and then for age, sex and case mix and were applied separately to children (≤ 14 years old) and adults (> 14 years old). Given the over-dispersion of the dependent variable, standard errors were scaled using the square root of deviance-based dispersion. Thus, the variance became more flexible, and the estimation bias of standard errors was minimised [15].

The SPSS 15.0 software package was used for statistical analyses. The significance of differences between groups was analysed using p-values, and p-values less than 0.05 were considered to indicate a significant difference.

## Results

One-fourth of the child population (26.2%) and almost one fifth of the adult population (17.9%) were immigrants (n = 69,067). While the age and sex distributions of immigrant and native children were similar, differences were observed among the adult population; for instance, there were fewer and younger women among immigrant adults (Table 1). The distribution of nationalities within the immigrant population is described in Table 2: the main immigrant flows arriving in Spain come from Latin America (34% of adult immigrants) and Eastern Europe (27% of adult immigrants).

**Table 1:** Age and sex distribution of the study population.

	Natives	Immigrants
**Sex**		

**Children**	**(n = 5,807)**	**(n = 2,058)**

Women	48.9%	47.9%

**Adults**	**(n = 50,220)**	**(n = 10,982)**

Women	53.4%	45.0%

**Age**		

**Children**	**(n = 5,807)**	**(n = 2,058)**

0-4	34.4%	35.8%
5-9	32.9%	33.1%
10-14	32.7%	31.1%

**Adults**	**(n = 50,220)**	**(n = 10,982)**

15-24	9.0%	16.8%
25-34	17.8%	38.0%
35-44	18.0%	27.3%
45-54	14.5%	12.3%
55-64	13.2%	3.7%
65-74	12.0%	1.2%
> = 75	15.5%	3.6%

**Table 2:** Distribution of nationalities within the immigrant population.

	Adults (n = 10,982)	Children (n = 2,058)
Latin America	33.9%	32.1%
Eastern Europe	26.8%	17.7%
Sub-Saharan Africa	16.7%	15.7%
North Africa	13.7%	16.0%
Asia	4.4%	6.6%
Western Europe & North America	4.3%	3.0%
Unknown	0.2%	8.9%

Among the study population, 59% of the adult immigrant population and 76% of the adult native population visited the health centre at least once during the study year; among the child population, these figures were 74% and 84%, respectively.

**Figure 1:**
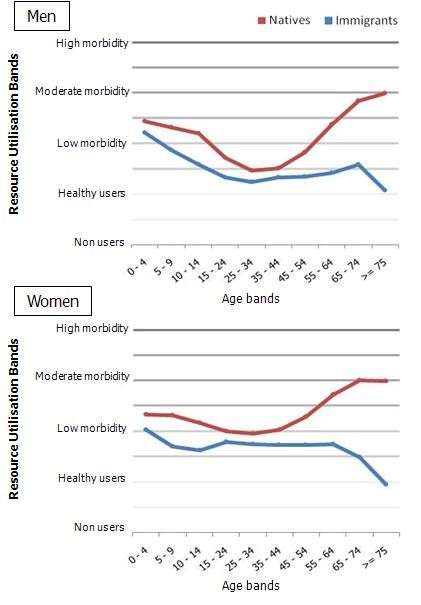
**Mean Resource Utilisation Band scores in immigrant and native populations by age band and sex**.

**Figure 2:**
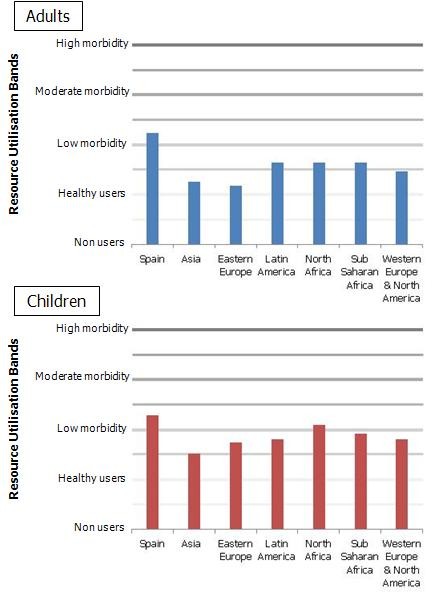
**Age- and sex-adjusted mean Resource Utilisation Band scores in adults and children by nationality**.

When analysing the average morbidity burden of the study population (measured by RUBs) lower levels are systematically observed among the immigrant population (Figure 1). Immigrants from Eastern Europe and Asia show the lowest levels of morbidity burden both within the adult and child population (Figure 2).

The age and sex adjusted mean number of total annual consultations was significantly lower among the immigrant population compared to the native population, with almost 2 fewer annual visits per child and 4 fewer annual visits per adult (Table 3).

**Table 3:** Age- and sex-adjusted mean number of annual consultations per 1,000 individuals by PC service and type of visit.

	Natives	Immigrants
**PC service**		

**Children**		

Paediatrics	6,214*	4,612*
Nursery	1,448	1,451
Diagnostic tests ^a^	118*	140*
Physiotherapy	1	1
Social work	3	7
Odontostomatology	79	76
Midwifery	---	---
Total	7,873*	6,304*

**Adults**		

Family medicine	5,252*	3,274*
Nursery	2,539*	763*
Diagnostic tests ^a^	639*	435*
Physiotherapy	78*	23*
Social work	25	21
Odontostomatology	65	61
Midwifery	55*	63*
Total	8,672*	4,654*

**Type of visit**		

**Children**		

Demanded visit	5,264*	4,172*
Emergency visit	1,173*	665*
Programmed visit	1,411	1,461
Home visit	25*	6*

**Adults**		

Demanded visit	5,341*	2,932*
Emergency visit	715*	558*
Programmed visit	2,267*	1,117*
Home visit	318*	42*

* Statistically significant differences between natives and immigrants (p < 0.05)

^a ^Diagnostic tests include those available in PC centres such as lab tests, ECGs, spirometry, ambulatory blood pressure monitoring, Holters, echography and glycaemia tests

Similar results were obtained when considering specific PC health services: significantly fewer visits were registered among immigrant adults for diagnostic tests and nursery, physiotherapy and midwife services. On the other hand, the number of annual consultations for diagnostic tests was slightly higher among immigrant children compared to native children (22 more annual visits per 1,000 children, p < 0.05) (Table 3).

When considering the type of visit, a significantly lower number of consultations was still observed among both child and adult immigrants, except in the case of programmed visits for which no statistical differences were detected between immigrant and native children (Table 3).

After adjusting for morbidity burden, the differences in PC visits almost disappeared. The incidence rate ratios (IRRs) for the total number of consultations among children increased from 0.79 to 0.94 (with the reference category being 'native'), although the difference in PC visits was still significant. For the adult population, the IRR after adjusting for case mix was precisely 1.0 (Table 4). Visits to nurses were still significantly lower among immigrant adults, even when considering their morbidity burden (IRR = 0.71, p < 0.05). In contrast, visits for diagnostic tests were significantly higher among both child and adult immigrants after adjusting for case mix (IRR = 1.77 and IRR = 1.21, respectively, p < 0.05).

**Table 4:** Incidence rate ratios and 95% confidence intervals of PC consultations in the immigrant vs. native population by PC health service and type of visit.

	Children		Adults	
	**IRR**^**1**^	**IRR**^**2**^	**IRR**^**1**^	**IRR**^**2**^

**PC health service**				

Paediatrics	0.73* (0.72 - 0.75)	0.89* (0.87 - 0.91)	---	---
Family medicine	---	---	0.76* (0.75 - 0.77)	1.03* (1.02 - 1.04)
Nursery	0.99 (0.95 - 1.03)	1.08* (1.03 - 1.12)	0.45* (0.44 - 0.46)	0.71* (0.68 - 0.73)
Diagnostic tests ^a^	1.33* (1.16 - 1.53)	1.77* (1.53 - 2.04)	0.92* (0.89 - 0.95)	1.21* (1.17 - 1.25)
Total	0.79* (0.78 - 0.81)	0.94* (0.91 - 0.96)	0.73* (0.72 - 0.74)	1.00 (0.99 - 1.01)

**Type of visit**				

Demanded visit	0.78* (0.77 - 0.80)	0.95* (0.93 - 0.97)	0.71* (0.70 - 0.72)	0.97* (0.96 - 0.98)
Emergency visit	0.56* (0.53 - 0.60)	0.68* (0.64 - 0.72)	0.90* (0.87 - 0.92)	1.20* (1.17 - 1.23)
Programmed visit	1.02 (0.97 - 1.06)	1.10* (1.05 - 1.14)	0.71* (0.69 - 0.72)	0.98 (0.95 - 1,00)
Home visit	0.25* (0.14 - 0.44)	0.29* (0.16 - 0.52)	0.26* (0.23 - 0.31)	0.41* (0.35 - 0.47)

* Statistically significant differences between natives and immigrants (p < 0.05)

Reference category: 'native'

IRR1: adjusted for age and sex

IRR2: adjusted for age, sex and case mix

^a ^Diagnostic tests include those available in PC centres such as lab tests, ECGs, spirometry, ambulatory blood pressure monitoring, Holters, echography and glycaemia tests

When considering the type of visit, differences between immigrants and the native population also disappeared after adjusting for morbidity burden, except for home visits, which remained remarkably lower among immigrants compared to the native population (IRR = 0.29 in children, IRR = 0.41 in adults, p < 0.05). The age, sex and case mix-adjusted IRRs for emergency PC visits remained <1.0 for children (IRR = 0.68, p < 0.05) but were >1.0 for adults (IRR = 1.20, p < 0.05).

## Discussion

The present study was carried out in the context of a national health system offering universal and free coverage, and the results confirm that PC utilisation is lower among immigrants compared to the native population. Moreover, our results corroborate the observation that immigrant populations have a lower morbidity burden and reinforce the so-called 'healthy migration effect' previously described [16-19]. Thus, differences in PC utilisation patterns decrease when considering the morbidity burden.

Using administrative data to objectively monitor the use of PC services by the immigrant population is increasingly important to host countries. It aids in the assessment of inequalities in the use of PC services and eventual access problems [6]. Unlike studies based on heath surveys, data collected from PC centres offer information on the true utilisation of PC services by the entire population, thus avoiding sampling or response biases. In this particular study, data were assessed from more than 69,000 individuals.

Contrary to certain stereotypes that immigrants make excessive use of health services [20], our study provides evidence for lower use of PC services by both child and adult immigrants after adjusting for age and sex. This finding held true for all types of visits except in the case of programmed visits for children. These results confirm the findings of previous studies in which lower use of PC services by immigrants was observed both in Spain [1,21] and in other developed countries [22-26].

We chose the ACG System to measure the morbidity burden because of its acknowledged validity and extensive use as a risk adjustment technology and because it can be constructed from commonly available administrative data. Based on these data, it develops a composite measure of morbidity burden estimated from a mixture of conditions experienced over a defined time interval [6]. According to the data provided by the ACG System, the health status of immigrants in our study was better than that of native citizens. These findings are consistent with the previously described 'healthy migration effect' [16-18,27]. The 'healthy migration effect' is a consequence of a self-selection process that excludes ill and disabled individuals and includes those with high capacities and personal motivations. Migratory processes favour the success of individuals with enhanced skills in terms of education, work and communication [28]. This healthier status of immigrants somehow justifies the lower PC utilisation seen among them. In this study, the differences in PC utilisation patterns almost disappeared among the adult population (IRR = 1.00, p > 0.05) and decreased considerably among children (IRR = 0.94, p < 0.05) after adjusting for case mix. These results suggest that for a given morbidity burden, there are no differences in PC utilisation patterns between immigrants and the native population. Health care for non-EU foreign nationals in Spain is regulated by Article 12 of Organic Law 4/2000 of 11^th ^January regarding the rights and freedoms of foreign nationals living in Spain and their social integration [10], which guarantees health care to foreign nationals regardless of their residency status under the same conditions as native Spaniards. This point may explain our results.

Interestingly, the mean number of routine diagnostic tests was 77% higher among immigrant children compared to native children (IRR = 1.77, p < 0.05). This finding could be due to preventive medical checkups carried out on immigrant children upon arrival in the host country [29,30]. In addition, it could indicate that GPs lack self-confidence when clinically evaluating these patients due to communication problems derived from language difficulties.

While the age, sex and case mix adjusted mean number of emergency visits was 30% lower among immigrant children (IRR = 0.68, p < 0.05), the immigrant adult population made greater use of such services (IRR = 1.2, p < 0.05). Other studies have highlighted the association between the use of the PC emergency service and work conditions among immigrants. The PC emergency service is an extension to GPs' normal schedule available until 9pm, Monday through Saturday. Difficulties in making work schedules compatible with the visiting hours of PC health centres have been suggested to result in higher usage of PC emergency services among immigrants [31]. It could be that the higher usage of PC emergency services makes up for the lower number of demanded visits (IRR = 0.97, p < 0.05) and home visits (IRR = 0.41, p < 0.05) among the immigrant adult population.

As for home visits, there is an evident lower use of such service by both adult (IRR = 0.41, p < 0.05) and child (IRR = 0.29, p < 0.05) immigrants which is a clear consequence of lack of information. Thus, improvements are still needed regarding the provision of information on health services access conditions.

One of the main limitations of the present study derives from the lack of available socioeconomic indicators. Given the absence of this information in administrative databases, we were unable to adjust for this characteristic, which has previously been found to explain a high degree of variability in the use of healthcare services [32]. Nevertheless, the consequences of this limitation might have been mitigated by the fact that all three PC centres included in this study belonged to areas with a similar socio-economic level.

The fact that we did not carry out an analysis that stratified the immigrant population according to country of origin suggests that we might have missed some interesting findings. There is evidence indicating that variations exist among different ethnic groups in terms of health care utilisation [32,33]. Such stratification was not possible in our study given the limited size of the immigrant population but should be considered in the future. Nevertheless, the majority of immigrants come from low-income countries (e.g., Latin America and Eastern Europe) and are categorised as economic immigrants. Immigrants from high-income countries (e.g., Western Europe and North America) represent a minority group (4.3% of adult immigrants and 3.0% of child immigrants). Immigrants from high-income countries have a legal status similar to that of the Spanish-born population, and their socioeconomic status is above the national average [34].

Another potential limitation of this study derives from the quality of the diagnostic information registered in the electronic medical records since the completeness and accuracy of data entry relies on the enthusiasm and idiosyncrasies of individual GPs [35]. Medical record data contains some errors and omissions, but there have been large improvements in its quality in recent years. As a result, this source of information is increasingly being used for epidemiological research in the Spanish context [36]. Moreover, a sensitivity analysis was carried out using data from two of the three PC health centres that were known to have received training sessions to encourage and improve the quality of morbidity coding among GPs. Neither the distribution of ACG frequency nor the associations observed in our study were significantly modified. In fact, the 'healthy migration effect' shown in the present study was reinforced given that the differences in the morbidity pattern of immigrants versus that of the native population became wider. Furthermore, the case mix of patients according to ACGs was consistent with that reported in other studies [37,38].

Finally, although all immigrants have the right to request a health card identifier regardless of their legal status and provided that they are registered in the local population census, it may occasionally occur that they do not seek such service for fear of deportation (i.e., irregular immigrants) or due to lack of information [39]. In such cases, it is possible that the same health card identifier might be used by various immigrants who attend PC health centres and use the card of a relative or a friend. The aforementioned problem could result in overestimation of the use of PC services by immigrants compared to the native population, thus increasing the differences found in this study. In other words, we believe that the consideration of this bias would not alter our findings.

Future research should focus on a comprehensive analysis of the use of health services by the immigrant population. This would entail integrating different data sources from different levels of care. In Spain, greater use of hospital emergency services by immigrants has been reported by several authors [40,41], although others have ruled out this hypothesis [42]. The use of specialist care has been shown to be lower among immigrants [43], and similar trends have been highlighted concerning the level of double (public-private) coverage [44]. Our results indicate that there seems to be a shift from demanded visits (during PC visiting hours) towards PC emergency visits among the adult immigrant population. Investigating whether this tendency goes beyond the PC level would lead to important findings regarding health care commissioning.

Also, this type of studies need to be reproduced with data from other urban and rural public healthcare settings in such a way that conclusions can be extended to the regional or even national level.

We did not have access to immigrants' dates of arrival in Spain. Although the increase in the immigrant population is a relatively recent phenomenon in Spain, the evolution of their patterns of health services utilisation and morbidity over time is worth studying. Several authors have shown how the health status of immigrants becomes poorer with time due to progressive deterioration of their lifestyles [25,45].

The conclusions related to the 'healthy migration effect' presented in this study are based on information regarding health conditions obtained from GPs' electronic medical records ('attended' morbidity). Research strategies should be designed to study differences in the objective and representative 'non-attended' morbidity burden of both native populations and immigrants.

## Conclusions

In Spain, the immigrant population makes lower use of PC services than the native population after adjusting the consultation rates for age and sex. When considering the morbidity burden, however, these differences decrease significantly. The present results reinforce the 'healthy migration effect' and discount the existence of differences in PC utilisation patterns between the immigrant and native populations in Spain. Studies of this type need to be carried out in the coming years to verify whether the health system is adequately responsive to the changing morbidity burden and health care needs of the immigrant population.

## Competing interests

The authors declare that they have no competing interests.

## Authors' contributions

LGF and APT generated the research question. BPP and DBB carried out the statistical analyses. ACL, LGF, RMC and APT participated in the interpretation and discussion of results. ACL and LGF contributed to the drafting of the manuscript. ACL coordinated the writing of the contribution. All authors read and approved the final manuscript.

## Pre-publication history

The pre-publication history for this paper can be accessed here:

http://www.biomedcentral.com/1471-2458/11/432/prepub
